# Fur4‐mediated uracil‐scavenging to screen for surface protein regulators

**DOI:** 10.1111/tra.12815

**Published:** 2021-09-23

**Authors:** Katherine M. Paine, Gabrielle B. Ecclestone, Chris MacDonald

**Affiliations:** ^1^ York Biomedical Research Institute and Department of Biology University of York York UK

**Keywords:** cell surface membrane proteins, endosomes, membrane trafficking, secretory pathway, yeast genetics

## Abstract

Cell surface membrane proteins perform diverse and critical functions and are spatially and temporally regulated by membrane trafficking pathways. Although perturbations in these pathways underlie many pathologies, our understanding of these pathways at a mechanistic level remains incomplete. Using yeast as a model, we have developed an assay that reports on the surface activity of the uracil permease Fur4 in uracil auxotroph strains grown in the presence of limited uracil. This assay was used to screen a library of haploid deletion strains and identified mutants with both diminished and enhanced comparative growth in restricted uracil media. Factors identified, including various multisubunit complexes, were enriched for membrane trafficking and transcriptional functions, in addition to various uncharacterized genes. Bioinformatic analysis of expression profiles from many strains lacking transcription factors required for efficient uracil‐scavenging validated particular hits from the screen, in addition to implicating essential genes not tested in the screen. Finally, we performed a secondary mating factor secretion screen to functionally categorize factors implicated in uracil‐scavenging.

## Graphical abstract

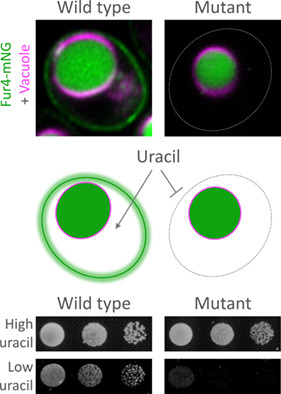

The uracil permease Fur4 localises to the yeast plasma membrane to uptake uracil from the extracellular environment (upper). Mutants with reduced surface levels of Fur4 have diminished capacity to uptake uracil (middle), and consequently exhibit growth defects specifically in low uracil media (lower). This uracil‐dependent growth phenotype was used to screen a library of haploid deletion mutants to reveal a large cohort of factors directly and indirectly associated with membrane trafficking.

## 1 INTRODUCTION

Cell surface membrane proteins are regulated by a variety of overlapping and often co‐regulated membrane trafficking pathways. Surface cargoes are co‐translationally imported into the endoplasmic reticulum (ER)^1^ before transiting the secretory pathway to the plasma membrane (PM).^2^ Surface proteins are internalized via clathrin‐mediated endocytosis, followed by recycling back to the PM or entering the lysosomal degradation pathway.^3^ These pathways allow surface localization and activity of myriad proteins to be precisely controlled to meet cellular demands, for example, during the cell cycle or in response to reduced nutrient availability. However, these pathways remain incompletely characterized. The budding yeast system has been useful to discover and define membrane trafficking mechanisms. Yeast cells uptake nutrients from their external environment by various transporters that localize to the PM, such as transporters for sugars, metal ions and vitamins.^4^, ^5^, ^6^ Amino acids and nucleobases are also actively transported into yeast cells via permeases, for example, Gap1 broadly uptakes amino acids,^7^ Mup1 uptakes methionine^8^ and Fur4 uptakes uracil.^9^ Permease activity can be controlled by changes in transporter expression, the rate of turnover by ubiquitin‐mediated vacuolar degradation, in addition to spatiotemporal control between eisosomes and other regions of the PM.^10^, ^11^, ^12^, ^13^, ^14^ To develop an assay that reports on nutrient uptake via surface transporters, we focussed on the uracil permease Fur4,^9^ which is controlled by all the above‐described trafficking pathways and regulatory mechanisms. For example, the presence of uracil downregulates expression of *FUR4* ^15^, ^16^ while also triggering endocytosis and Rsp5‐mediated ubiquitination and degradation of Fur4.^17^, ^18^ Furthermore, Fur4 activity is regulated in response to metabolic stress via storage in eisosomes.^19^, ^20^, ^21^ Beyond this, Fur4‐mediated uptake of uracil might be considered particularly important for many lab strains that cannot synthesize uracil biosynthetically, because of disruption of the orotidine‐5′‐phosphate decarboxylase *URA3* gene (eg, *ura3‐52* or *ura3∆*), which causes the useful selection characteristics of auxotrophy and resistance to 5‐fluroorotic acid.^22^ Indeed, many genome‐wide libraries^23^, ^24^, ^25^, ^26^, ^27^, ^28^, ^29^ have been created from parental *ura3∆* strains.^30^ We therefore chose Fur4‐mediated uptake to develop a simple and cost‐effective growth assay that indirectly reports on the surface‐mediated uptake of uracil by the Fur4 transporter, which we used to screen a haploid library of deletion mutants for factors that regulate Fur4 trafficking.

## 2 RESULTS AND DISCUSSION

### 2.1 A Fur4‐activity‐based growth assay

**FIGURE 1:**
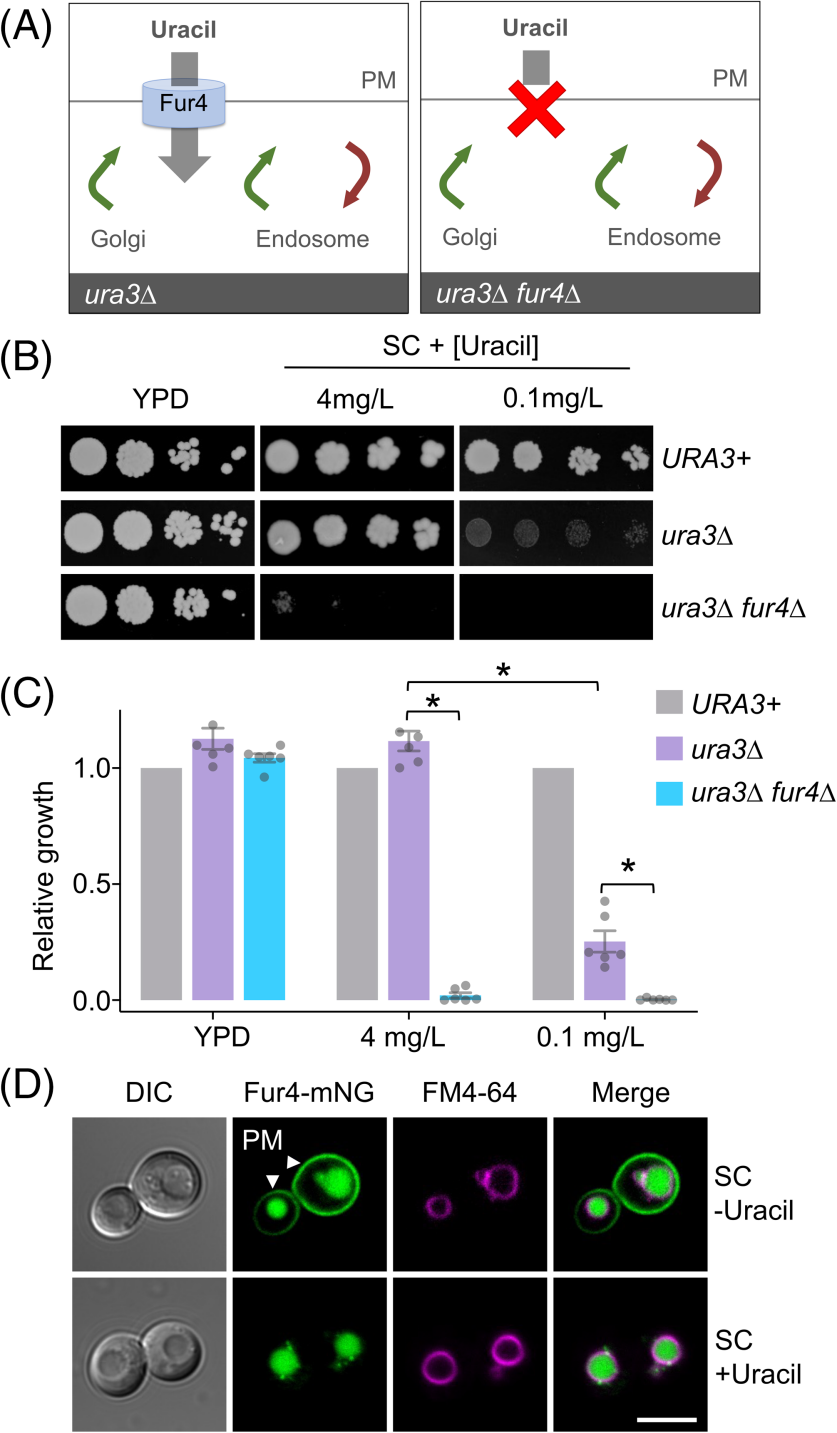
Low‐uracil growth relies on the Fur4 transporter. (A) Schematic illustration showing uracil‐auxotroph cells (*ura3∆*) with (left) and without (right) the uracil permease Fur4. (B) Indicated yeast strains were grown to mid‐log phase before plating on rich (YPD) and synthetic complete (SC) media containing either 4 or 0.1 mg/L uracil. (C) Quantification of yeast growth from (B), asterisks (*) indicate Student *t* test comparisons *P* = <.001. (D) Wild‐type cells expressing Fur4‐mNeonGreen (Fur4‐mNG) from its endogenous promoter were labelled with FM4‐64 for 1‐hour, grown to mid‐log phase in SC‐Ura media (upper) or in the presence of 40 μg/mL uracil for 1 hour (lower) prior to confocal microscopy. Arrows indicated plasma membrane (PM), scale bar = 5 μm

The uracil permease Fur4 is dispensable for growth in rich media but is critically required when uracil‐auxotroph cells are grown in synthetic defined media containing replete (4 mg/L) uracil (Figure 1A–C). Importantly, the robust Fur4‐dependent growth of BY4742 cells, which harbour a *ura3∆* mutation,^30^ herein referred to as wild type, corresponds to the concentration of available uracil. There is a significantly reduced rate growth when wild‐type cells are grown in media containing 0.1 mg/L uracil, but Fur4‐dependent uracil‐scavenging supports growth (Figure 1B,C). Fur4 localization has been previously shown to respond to extracellular uracil,^16^ and we confirm steady state surface localization of Fur4 tagged with mNeonGreen (mNG) is redistributed to FM4‐64 stained vacuoles following 1‐hour of uracil addition to the media (Figure 1D). Collectively these results show that the activity of the uracil‐sensitive permease Fur4 correlates with cellular growth in limited uracil conditions.

**FIGURE 2:**
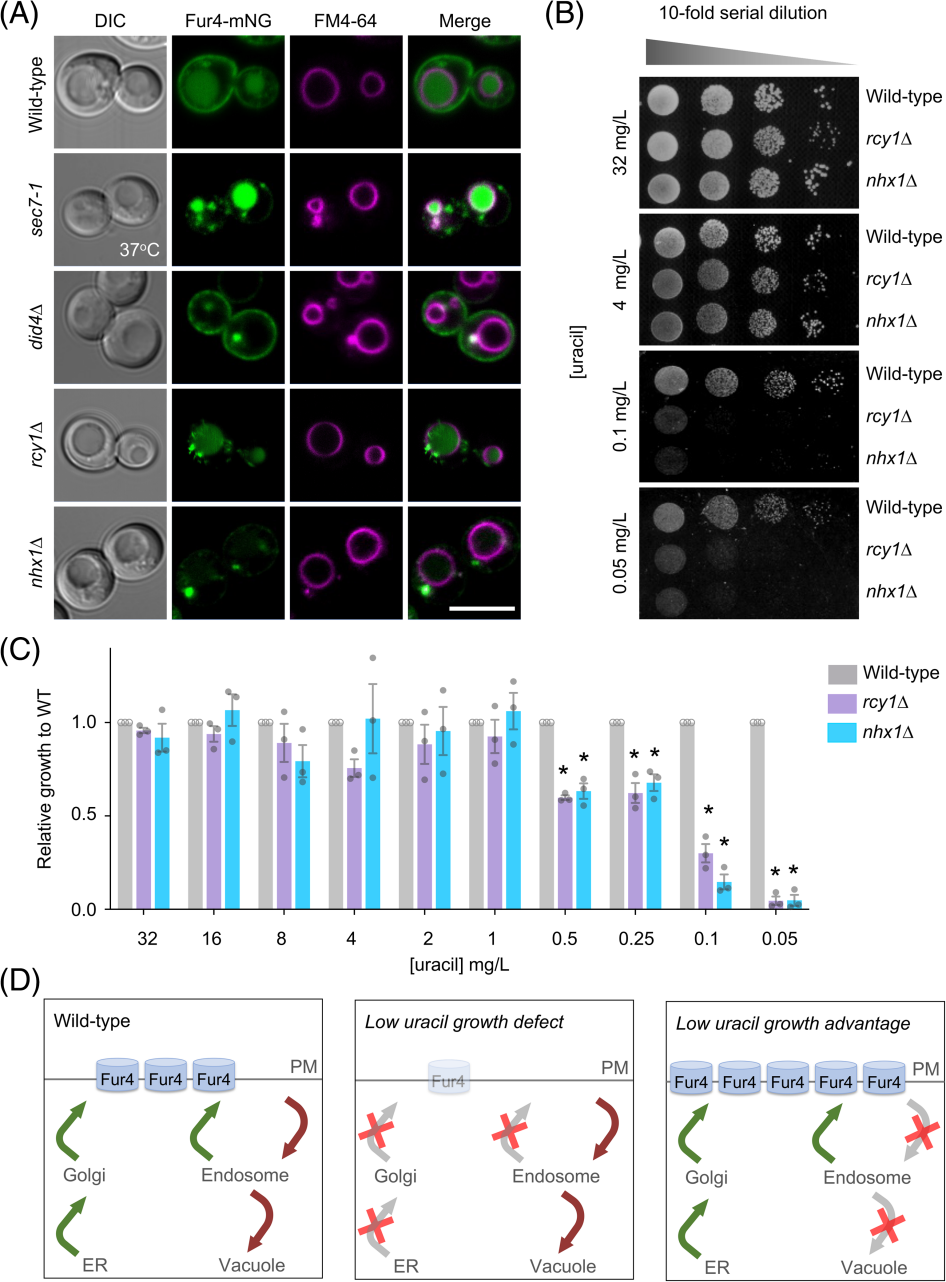
Surface localization of Fur4 is required for growth in low uracil. (A) Vacuoles from indicated strains expressing Fur4‐mNeonGreen (mNG) were labelled with FM4‐64 prior to confocal imaging. (B) Wild‐type, *rcy1∆* and *nhx1∆* cells grown to mid‐log phase were then spotted in a 1 in 10 serial dilution onto plates titrated with indicated concentrations of uracil and grown at 30°C for 3 days. (C) Growth of strains from (B) were quantified, asterisks (*) indicate Student *t* test comparisons of mutants with wild‐type cells, *P* = <.005. (D) Schematic diagrams showing the predicted effects on Fur4 following different trafficking pathway perturbations. Scale bar = 5 μm

To test if low‐uracil‐specific growth could be used to screen for membrane trafficking factors that influence Fur4 surface levels, we next compared Fur4‐mNG localization in wild‐type cells and mutants that mislocalize Fur4 (Figure 2A). As expected, a temperature‐sensitive *sec7‐1* allele, which disrupts activity of the Sec7 Arf‐exchange factor required for transit through the Golgi,^31^, ^32^ inhibits trafficking of Fur4 through the secretory pathway, with Fur4‐mNG accumulating in intracellular puncta instead of the PM. Fur4‐mNG localization is also affected in *did4∆* (*vps2∆*) ESCRT mutants,^33^ that do not permit vacuolar sorting and Fur4‐mNG instead accumulates in endosomes. However, uracil‐scavenging is likely efficient in *did4∆* cells as significant Fur4‐mNG recycling back to the PM is observed, unlike Mup1‐GFP that is trapped by Snf7‐oligomers.^34^ In contrast, surface levels of Fur4‐mNG was greatly reduced in both *rcy1∆* or *nhx1∆* mutants (Figure 2A), which lack factors required for endosomal recycling.^35^, ^36^, ^37^ Therefore, we compared growth of *rcy1∆* or *nhx1∆* mutants with wild‐type cells on plates of varying uracil concentrations. There was no statistically significant difference in growth between wild‐type and trafficking mutant cells in the range of 1 to 32 mg/L uracil, but at lower uracil concentrations the *rcy1∆* and *nhx1∆* mutants, which have reduced surface Fur4, exhibit a low‐uracil‐specific growth defect (Figures 2B,C and S1a, Supporting Information). Although significant defects were observed at 0.5 and 0.25 mg/L uracil, we selected 0.1 and 0.05 mg/L uracil for scavenging conditions, as wild‐type cells grow efficiently but *rcy1∆* or *nhx1∆* both show dramatically reduced growth. Low‐uracil‐specific growth defects were not observed in *did4∆* cells, demonstrating the PM levels observed by microscopy are sufficient to scavenge uracil, or *sec7‐1* cells at permissive temperature, but lethality at 37°C was confirmed (Figure S1b). We tested this concept with another surface‐localized nutrient transporter, the methionine transporter Mup1 grown in methionine auxotroph (*met15∆*) cells but no methionine concentration that supports growth could distinguish trafficking mutants (Figure S2). We assume differences in steady state surface levels, substrate affinity and uptake pathways^8^ account for this.

### 2.2 A genome‐wide screen for uracil‐scavenging mutants

**FIGURE 3:**
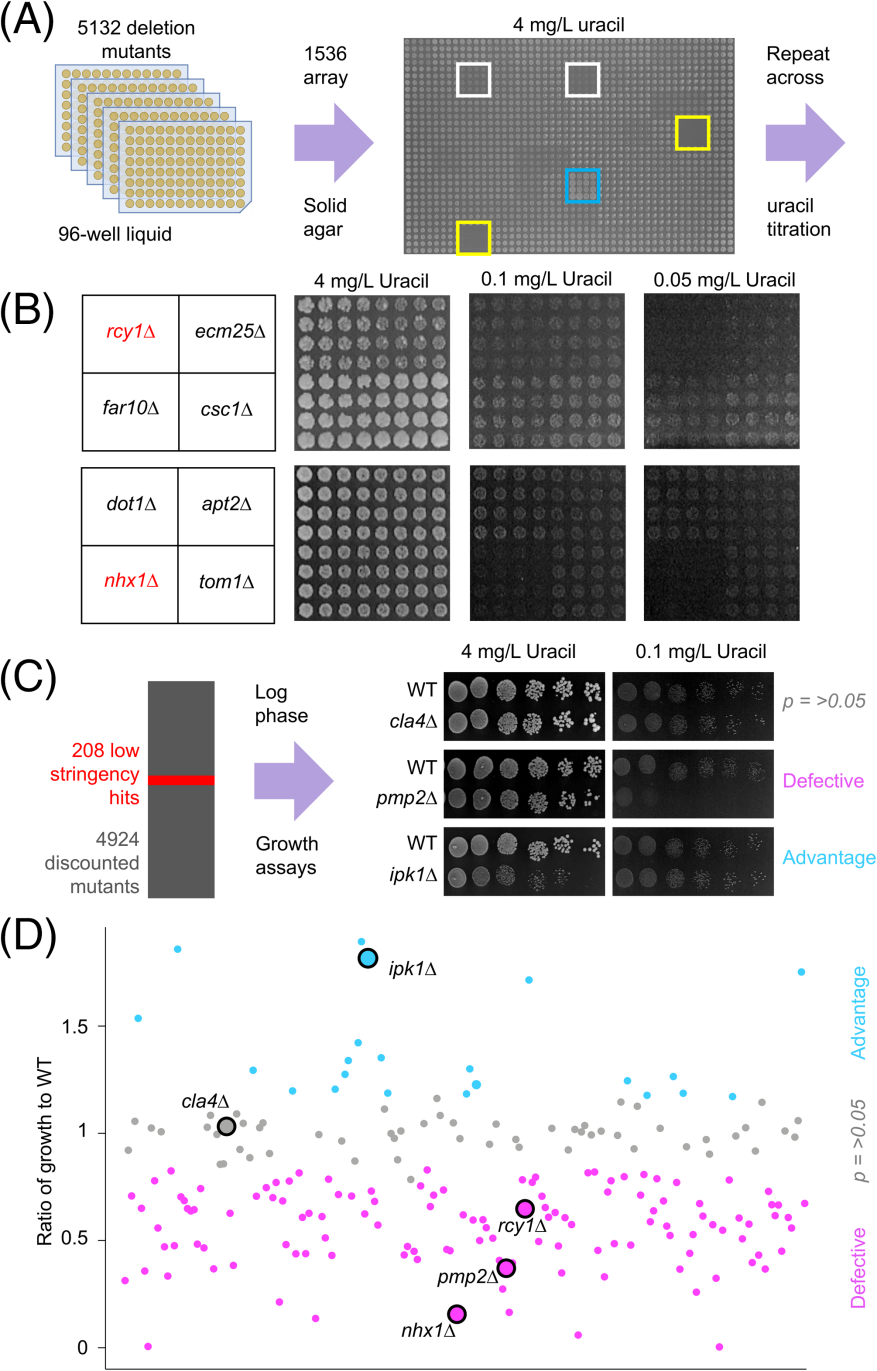
A genetic screen for mutants that affect uracil‐scavenging. (A) Yeast strains grown overnight in 96‐well plates were diluted 20‐fold in water then replicated 16 times onto solid agar plates containing varying uracil concentrations. An example 4 mg/L uracil plate shows identifier wells (yellow), alongside strains with defective (white) and accelerated (blue) growth. (B) Example of screen data showing indicated mutants grown on media containing 4, 0.1 and 0.05 mg/L uracil, including known Fur4 trafficking mutants (red). (C) The screen identified 208 candidates that were subsequently grown to mid‐log phase and spotted out over a 6‐step, 10‐fold serial dilution onto high (4 mg/L) and lower (0.1 and 0.05 mg/L) uracil containing media. Uracil‐related cellular growth relative to wild‐type was quantified and categorized as defective (eg, *pmp2∆*), advantageous (eg, *ipk1∆*) or not significantly altered (eg, *cla4∆*). (D) Ratio of relative growth between 4 and 0.1 mg/L uracil from (C) was plotted for all candidates

We hypothesized that mutants with growth similar to wild‐type cells in replete uracil but differences specifically at low uracil could be used to identify mutants from a nonessential haploid deletion library^28^ with perturbed surface levels of Fur4 (Figure 2D). Cultured yeast strains representing 5132 different mutants were diluted and spotted out (16 replicates of each) on to solid agar media containing replete (4 mg/L) and limited (0.1 and 0.05 mg/L) uracil concentrations (Figure 3A). As expected, mutants with growth differences in uracil‐replete media were observed, but the screen was specifically focused on differences in growth between high and low uracil concentrations. This is exemplified by the mutants used to calibrate the assay, *rcy1∆* and *nhx1∆*, which both exhibit growth defects specifically in low uracil when compared with neighbouring mutants (Figure 3B). A low stringency scoring system was used to identify 208 null mutants for follow‐up analysis. Candidates were grown to mid‐log phase then serially diluted and spotted on 4, 0.1 and 0.05 mg/L uracil media. Growth was quantified for the 208 mutant strains compared to a wild‐type control from the same plate, and then values were used to compare growth across uracil concentrations (Table S1). Statistical comparisons of mutants from these optimized growth assays revealed 58 mutants, such as *cla4∆* (Figure 3C), that did not show a significant difference in growth compared to wild type. However, 126 mutants with significant growth defects specifically in uracil‐scavenging conditions were identified (Figure 3D), ranging from relatively subtle defects (eg, *vps74∆* at 0.1 mg/L = 0.78 ± 0.04) to extreme (eg, *vps3∆* at 0.1 mg/L = 0.004 ± 0.003). Furthermore, although the assay was calibrated for mutants with defective growth in low uracil, the screen identified 24 mutants with enhanced growth compared to wild type. We note many of these strains exhibit growth defects in uracil replete media, such as *ipk1∆* (Figure 3C), allowing for benefits to be observed at low uracil (Table S1).

### 2.3 Screen enriched for molecular complexes

**FIGURE 4:**
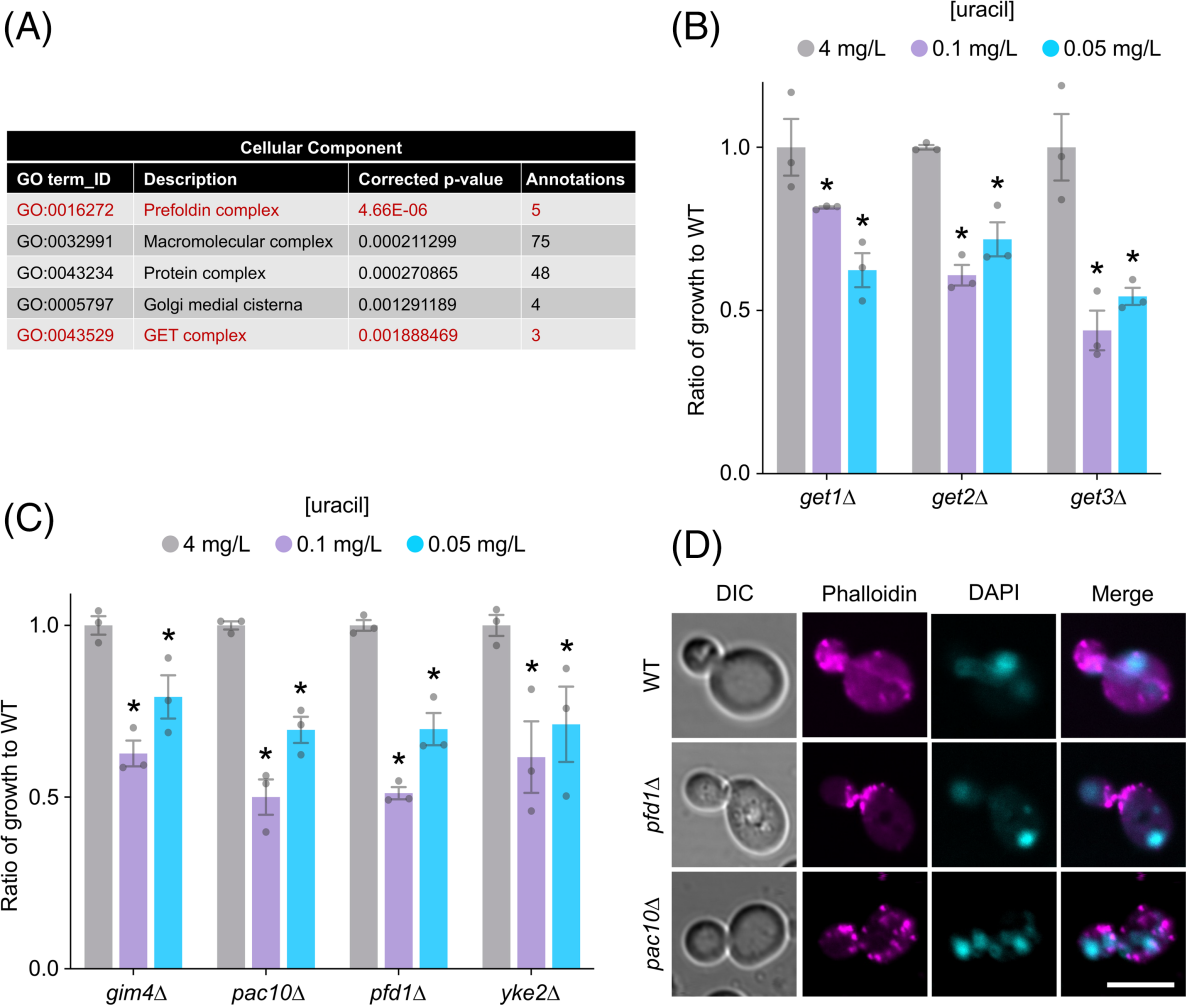
Low‐uracil screen identifies multi‐subunit complexes. (A) Gene ontology enrichment analysis for cellular component of the 150 factors identified from the screen. (B) Ratio of growth compared to wild‐type cells at 4, 0.1 and 0.05 mg/L uracil for GET complex mutants: *get1∆*, *get2∆* and *get3∆*. Asterisks (*) indicate Student *t* test comparisons *P* = <.001. (C) Ratio of growth compared to wild‐type cells at 4, 0.1 and 0.05 mg/L uracil for indicated prefoldin complex mutants: *gim4∆*, *pac10∆*, *pfd1∆* and *yke2∆*. Asterisks (*) indicate Student *t* test comparisons *P* = <.01. (D) Confocal microscopy of wild‐type (WT) and prefoldin mutants: *pfd1∆* and *pac10∆* stained with actin dye Phalloidin‐594 and nuclear dye DAPI and fixed with 4% paraformaldehyde. Scale bar = 5 μm

A comparative orthologue search^38^ of uracil‐scavenging mutants identified 94 highly conserved genes, corresponding to 267 human orthologues associated with 87 distinct diseases (Table S2). Gene Ontology (GO) Slim terms were obtained^39^ and revealed a number of cellular component enrichments of molecular complexes (Figure S3), including the GET complex and the prefoldin complex (Figure 4A). The identification of multiple complex members suggests the screen was stringent and robust. For example, deletion of *GET1*, *GET2*, or *GET3* results in low‐uracil growth defects (Figure 4B). As the GET complex is required for sorting of tail‐anchored single‐pass membrane proteins,^40^ many of which are essential factors in secretory pathway trafficking, such as SNARE proteins,^41^ we presume the role of the GET complex in efficient surface trafficking of Fur4 is via an indirect membrane trafficking mechanism. Various prefoldin complex members were also identified as having defective growth specifically on low uracil (Figure 4C), implicating it as a potential regulator of Fur4 trafficking. This could be explained by prefoldin‐mediated assembly of cytoskeleton proteins.^42^, ^43^ Indeed, actin filament structures observed in wild‐type cells are absent in prefoldin mutant cells *pfd1∆* and *pac10∆* (Figure 4D), indicating impaired cytoskeletal function that could adversely affect correct trafficking of Fur4 to the surface. However, the prefoldin complex is also involved in transcriptional elongation,^44^ so its role in uracil‐scavenging could be less direct.

### 2.4 Screen enriched for trafficking and transcriptional machinery

**FIGURE 5:**
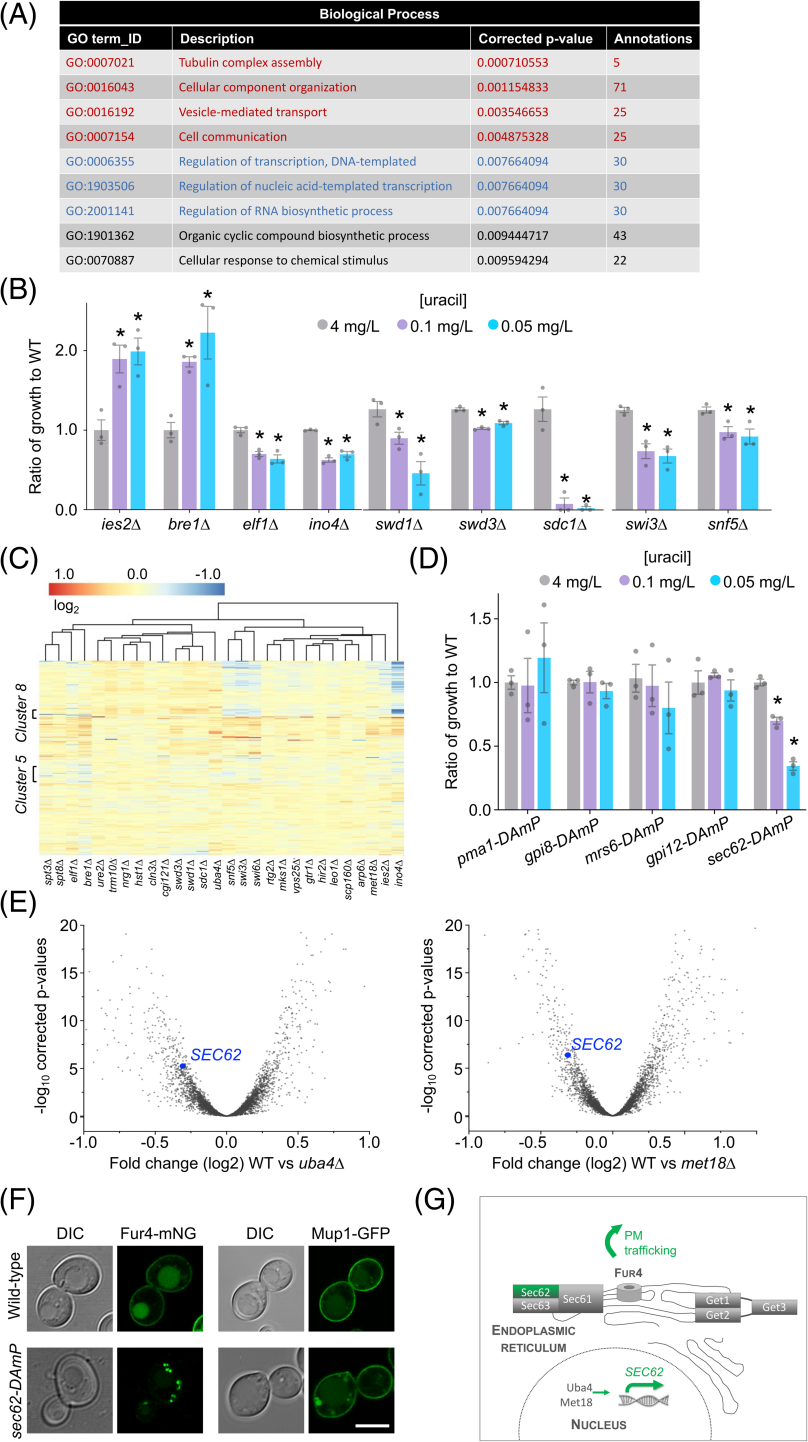
Trafficking screen enriched for transcriptional regulation. (A) Details of enriched annotations for gene ontology terms related to for biological processes for the 150 candidates identified in the screen. (B) Ratio of growth for selected transcription factor mutants identified in the screen compared to wild‐type cells at 4, 0.1 and 0.05 mg/L uracil. Asterisks (*) indicate Student *t* test comparisons *P* = <.05. (C) Hierarchical clustering and heat map of essential gene expression profiles in 28 different transcription factor null cells, coloured based on fold change. (D) Ratio of growth for indicated mutant strains compared to wild‐type (WT) cells grown on media containing 4, 0.1 and 0.05 mg/L uracil. Asterisks (*) indicate Student *t* test comparisons *P* = <.0001. (E) Volcano plots were constructed for log_2_ fold changes and their corresponding—log^10^ corrected *P*‐values for genes in microarray analyses comparing wild‐type cells to indicated mutants, with *SEC62* labelled (blue). (F) Airyscan microscopy of Fur4‐mNG and Mup1‐GFP expressed from their respective endogenous promoters in wild‐type and *sec62‐DAmP* cells. Scale bar = 5 μm. (G) Schematic diagram highlighting transcriptional and ER‐associated factors identified from the screen that contribute to efficient trafficking of Fur4 to the surface

GO enrichments for biological process revealed almost a third (44/150) of annotations were for machinery associated with membrane trafficking and signalling (Figure 5A and Table S3), including the blind identification of *rcy1∆* and *nhx1∆* mutants that were used to calibrate the assay (Figure 2). The other biological process significantly enriched was transcription, including three prefoldin subunit annotations (Figure 5A and Table S3). Physically interacting transcription factors were identified (Figure S3), such as members of the Complex Proteins Associated with Set1 (COMPASS) complex, Swd1, Swd3 and Sdc1^45^ alongside the Swi3/Snf5 pair^46^ among others that gave significant defects in low uracil (Figure 5B). We reasoned transcriptional regulators could be indirectly involved in Fur4 membrane trafficking, controlling gene expression of either essential genes not tested in the primary screen or mutants identified from the screen itself. To explore transcription factors (TFs) implicated in uracil‐scavenging, we assembled genome‐wide expression datasets for wild‐type cells vs 28 TF‐null mutants from a large‐scale microarray analyses.^47^ A matrix of all mutants showed high correlation of associated factors, such as known complex members (Figure S4). Cross‐referencing expression data for 1183 genes that are essential for viability (Table S4), followed by hierarchical clustering was used to generate a heat map of related gene expression changes (Figure 5C). Strains, such as *ies2∆*, *bre1∆* and *elf1∆* created distinct expression signatures, but others were similar, such as each of the COMPASS complex mutants *swd1∆*, *swd3∆* and *sdc1∆*. Particularly modulated clusters of genes were identified from this analysis (Table S5), including genes associated with membrane trafficking that we chose for experimental testing (Pma1, Gpi8, Mrs6, Gpi12 and Sec62). To achieve this, we performed uracil‐scavenging assays with strains containing Decreased Abundance by mRNA Perturbation (DAmP) cassettes at the 3′ UTR of each candidate.^48^ This analysis revealed *sec62‐DAmP* cells, which have very low protein levels of Sec62,^49^ have specific growth defects in low uracil (Figure 5D). *SEC62* expression was greatly reduced upon deletion of several TFs from the screen, for example, *uba4∆* and *met18∆* mutants (Figure 5E). The uracil‐scavenging defects in *sec62‐DAmP* cells can be explained in the context of reduced Fur4 trafficking to the surface, as shown by localization defects of Fur4‐mNG, and an unrelated transporter Mup1‐GFP (Figure 5F). Collectively, this example suggests that Uba4 and Met18 regulate expression of sufficient levels of Sec62, which are all required for proper trafficking of cargoes to the PM (Figure 5G).

### 2.5 Uncharacterized factors are controlled at the transcriptional level

**FIGURE 6:**
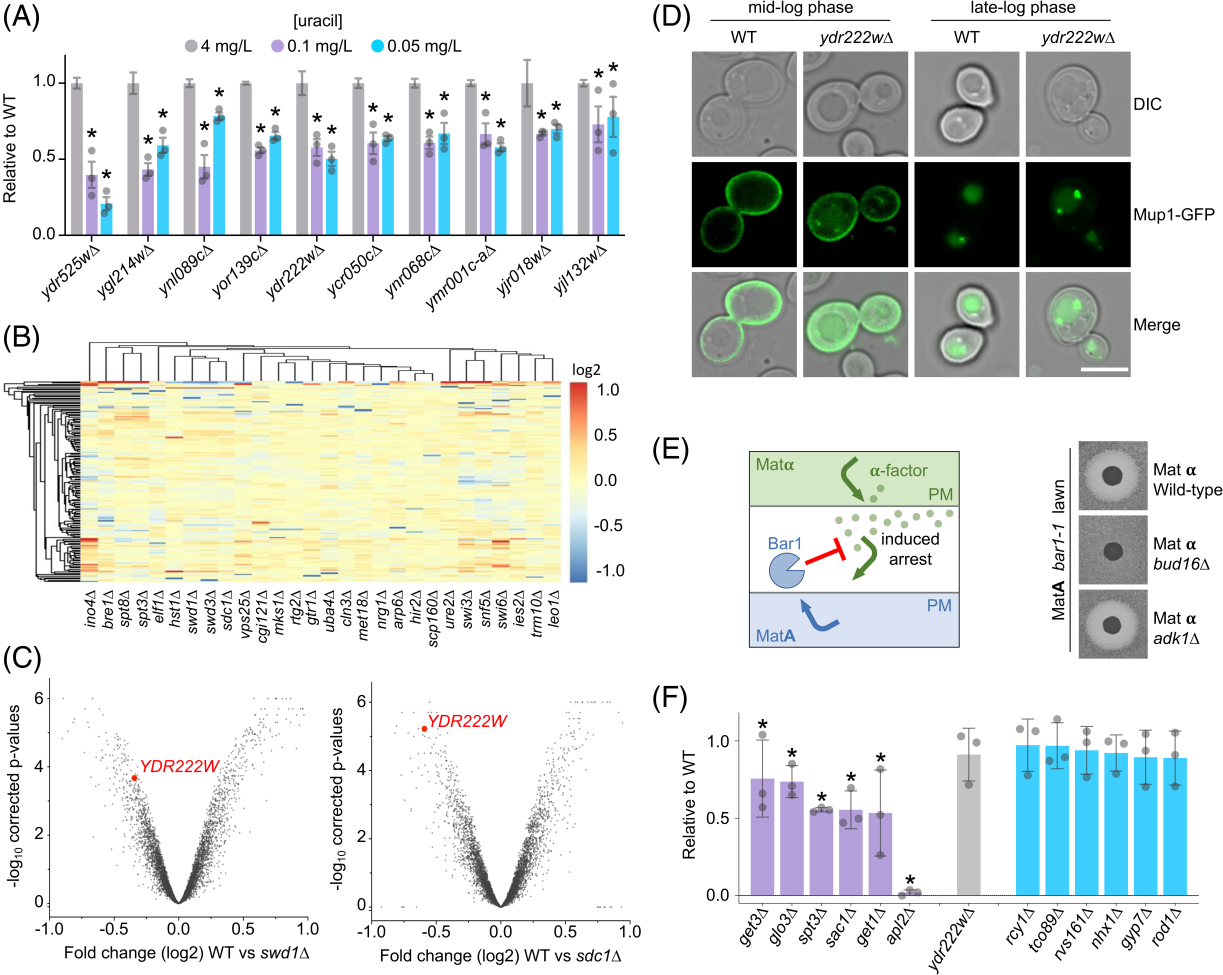
Previously uncharacterized proteins implicated in membrane trafficking. (A) Ratio of growth compared to wild‐type cells at 4, 0.1 and 0.05 mg/L uracil for indicated uncharacterized mutants identified in the screen. Asterisks (*) indicate Student *t* test comparisons *P* = <.001. (B) Hierarchical clustering of genes identified from the screen, plotting expression profiles as a heat map based on fold changes following deletion of 28 different transcriptional regulators. (C) Volcano plots constructed for log_2_ fold changes and their corresponding *P*‐values for genes in microarray analyses comparing wild‐type cells to *swd1∆* cells (left) and *sdc1∆* cells (right). Value for *YDR222W* is shown in each plot (red). (D) WT and *ydr222w∆* cells expressing Mup1‐GFP were imaged at mid‐log (OD_600_ = 1.0) and late‐log phase (OD_600_ > 2). Scale bar = 5 μm. (E) Schematic showing basis of α‐factor induced inhibition of growth of mutants from the primary screen on a lawn of *bar1‐1* MatA mutants (left), with representative examples shown (right). (F) Quantification of growth inhibition surrounding spots of Matα cells from screen described in (C), shown relative to wild‐type controls from same plate (*n* = 3). Asterisks (*) indicate Student *t* test comparisons *P* = <.1

The uracil‐scavenging screen identified 10 uncharacterized candidates (Figure 6A). In an effort to understand whether these were also regulated by the TFs from the screen, we used a similar approach to cross‐reference gene expression profiles of the TF‐deletion strains against the 150 genes identified in the screen. Again, hierarchical clustering revealed many gene profiles share signatures across different TF deletion experiments (Figure 6B). We were particularly intrigued by *ydr222w∆* mutants defective in growth from the screen (Figure 6A) as *YDR222W* was greatly downregulated in many of the transcription factor null mutants, including the *swi3Δ*, *spt3∆* and COMPASS complex mutants, but upregulated in other mutants not associated with low‐uracil growth (Figures 6C and S5). We find *ydr222w∆* mutants are defective in general surface protein trafficking, as the distinct Mup1‐GFP cargo exhibits mislocalization phenotypes at mid‐log phase and upon increased endocytosis via Sna3^50^ and Cos proteins^51^, ^52^ at late‐log phase (Figure 6D). This approach helps prioritize factors, like the uncharacterized protein Ydr222w, for follow‐up testing and can be used for essentially any genetic screen, even retrospectively, that identifies transcriptional regulators. In an effort to help functionally categorize all the 150 mutants from the screen, and to begin unravelling where uncharacterized factors like Ydr222w might function, a secondary screen was performed based on the trafficking of Alpha factor through the secretory pathway to induce cell cycle arrest on a lawn of Mat A yeast, which are sensitized to arrest by virtue of a mutation in the gene that encodes the Bar1 mating factor protease (*bar1‐1*). Alpha factor is synthesized and subjected to post‐translational modifications through the secretory pathway before being secreted.^53^ We hypothesized that mutants with defects in the secretory pathway leading to reduced alpha factor secretion would result in a reduction in the growth arrest response seen when Matα cells are spotted onto a lawn of MatA cells (Figure 6E). Conversely, mutants with no halo phenotype could act downstream of the PM, or indirectly, to explain uracil‐scavenging phenotypes. We observed mutants to have a reduction in halo size (eg, *bud16∆*) or a halo comparable to WT cells (eg, *adk1∆*). Mutants of proteins well characterized to act in the early stages of the secretory pathway such as GET‐complex nulls (discussed above) and *apl2∆* ^40^, ^54^ were seen to display a significantly reduced halo phenotype. While mutants of factors acting downstream of the secretory pathway in the endosomal system such as *rcy1∆* and *nhx1∆* ^35^, ^37^ were seen to display a halo phenotype not significantly different from WT (Figure 6F). Interestingly, uncharacterized mutant *ydr222w∆* gave a halo type not significantly different to WT cells. It might be the role of Ydr222w, which shares homology to the Svf1 protein implicated in survival pathways,^55^ functions at endosomes to regulate nutrient transporters in response to changes in extracellular nutrients.

### 2.6 Inducible tools to study surface proteins

**FIGURE 7:**
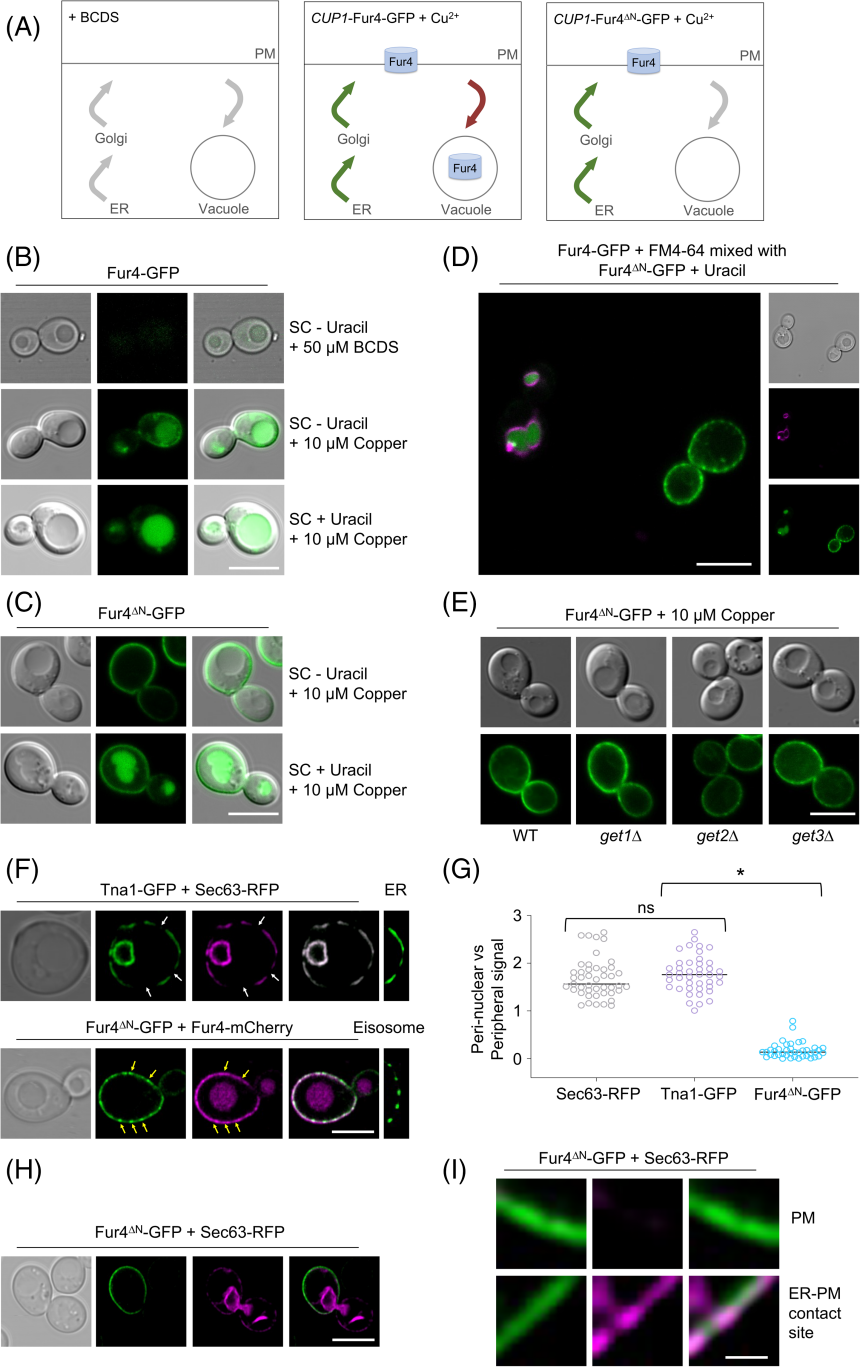
Inducible tools to study surface proteins. (A) Schematic diagram showing repression of the *CUP1* promoter in the presence of bathocuproine disculfonic acid (BCDS) (left), and the copper induced expression of Fur4‐GFP, which localizes to the PM and vacuole (middle) and Fur4^∆N^‐GFP that localizes exclusively to the surface. (B–D) Wild‐type cells transformed with *CUP1‐*Fur4‐GFP (B) and *CUP1*‐Fur4^∆N^‐GFP plasmids were grown under indicated conditions prior to fluorescence microscopy. A mixture of these cells, with vacuoles of Fur4‐GFP expressing cells first labelled with FM4‐64, were also imaged simultaneously (D). (E) Indicated cells expressing Fur4^∆N^‐GFP were grown to log‐phase followed by confocal microscopy. (F) Cells co‐expressing Tna1‐GFP and Sec63‐RFP (upper) or Fur4^∆N^‐GFP and Fur4‐mCherry (lower) were imaged by Airyscan microscopy. An example of Tna1‐GFP localized to the cortical ER (upper) and Fur4^∆N^‐GFP localized to eisosomes is included on right panels. White arrows indicate regions of the cortical ER that are not closely associated with the PM, yellow arrows indicate eisosomes. (G) The fluorescence signal of perinuclear ER was compared with peripheral signal (either cortical ER or PM) for Sec63‐RFP, Tna1‐GFP and Fur4^∆N^‐GFP. (H) Wild‐type cells expressing Fur4^∆N^‐GFP + Sec63‐RFP were grown to mid‐log phase and then imaged by Airyscan microcopy. (I) A zoomed in region of the periphery from (H) indicating Fur4^∆N^‐GFP localizing exclusively to the PM (upper) and membrane contact sites with the ER (lower)

In addition to fluorescently labelled Fur4‐mNG, and other surface cargoes affected by mutants from the screen, like Mup1‐GFP and the G‐protein coupled receptor Ste3 tagged with mCherry (Figure S6), for localization experiments to validate trafficking mutants, we created GFP tagged versions of both full length Fur4, or a mutant lacking its N‐terminal 60 residues under copper inducible *CUP1* promoter, to allow temporal control of trafficking (Figure 7A–D). We reasoned Fur4^∆N^‐GFP would be a suitable reporter for secretory pathway mutants, as it cannot be endocytosed,^18^ however localization in *get1∆*, *get2∆* and *get3∆* was indistinguishable from wild‐type cells (Figure 7E). This might suggest the GET complex exhibits a distinct function in uracil‐uptake. However, given the GET complex is known to be involved in secretory trafficking^40^ and in addition to all three members being identified from a blind screen for uracil‐scavenging, *get1∆ get2∆* and *get3∆* cells were also all defective in secretion of mating factor (0.53 ± 0.28, 0.80 ± 0.15 and 0.76 ± 0.24, respectively). Therefore, we favour the explanation that the uracil‐scavenging assay is sensitive enough to reveal a phenotype that is not apparent from steady state localization experiments. We did observe small amounts of signal at the ER of the truncated Fur4^∆N^‐GFP. To confirm that the peripheral Fur4^∆N^‐GFP signal was indeed the PM, and not the cortical ER, we imaged the ER marker Sec63‐RFP and GFP labelled versions of nicotinic acid permease Tna1, which traffics through the secretory pathway when tagged at the N‐terminus (Figure S7) but is retained in the ER when tagged at the C‐terminus.^26^ Unlike these ER proteins, Fur4^∆N^‐GFP signal is contiguous at the surface and localizes to eisosomes (Figure 7F), as previously documented.^21^ Furthermore, perinuclear signal of Fur4^∆N^‐GFP is very weak compared to the peripheral signal, unlike the ER localised proteins that exhibit the opposite phenotype with an intense signal at the perinuclear ER compared with the cortical ER (Figure 7G). Finally, we show clear distinction between Fur4^∆N^‐GFP at the PM and ER‐PM contact sites^56^ indicated by Sec63‐RFP (Figure 7H,I).

## 3 SUMMARY

In conclusion, we report a simple growth assay that indirectly reports on surface protein trafficking via nutrient transporter activity of uracil auxotroph yeast strains. The assay relies on comparison of growth efficiency of yeast cells on relatively high and low uracil media to infer the capacity of the Fur4 transporter to scavenge uracil required for growth. It is therefore cheap, simple and easy to perform at high throughput, as demonstrated by testing a haploid deletion library of over 5000 yeast strains. This genetic screen identified many novel candidates as potential Fur4 regulators and was particularly enriched for membrane trafficking and transcriptional machinery. By cross‐referencing essential genes and factors identified from the screen, with genome‐wide expression patterns in most of these transcriptional regulators, we were able to identify connections between TFs and the genes they regulate, both of which relate to uracil‐scavenging. As an example, our bioinformatics identified the essential gene *SEC62* and the uncharacterized gene *YDR222W*, as repressed in many of the TFs mutants identified from the screen. As proof of principle, we show experimentally that decreased expression of *SEC62* does, as expected, result in defects in surface protein trafficking. Similarly, we confirm a role for Ydr222w in surface protein trafficking, highlighting the discovery benefits in following transcriptional regulators identified for a given genetic screen. Indeed, we have recently identified an unexpected trafficking role for the Mig1 transcriptional regulator in endocytosis^20^ and endosomal recycling,^57^ suggesting the >20 candidates implicated in this study could also be explored functionally in the context of either global or cargo specific cargo trafficking mechanisms. Although we cannot exclude the possibility that mutants have indirect effects on uracil scavenging, for example via the biosynthetic or metabolic processes. However, as most genes were either enriched for membrane trafficking or can be functionally explained in the context of membrane trafficking, this suggests the bulk of mutants reported likely affect trafficking pathways used by Fur4. Furthermore, both factors prioritized by bioinformatics, Sec62 and Ydr222w, are shown to be required for proper sorting of fluorescently labelled cargoes. Our data support the idea that mutants with even subtle decreases in Fur4 at the PM can reveal a uracil‐scavenging phenotype. Therefore, we propose this uracil‐scavenging assay, used in combination with fluorescently tagged cargoes, the mating factor secretion assay and bioinformatic approaches all documented herein serve as useful tools to study surface protein trafficking, in addition to the mutants identified and characterized, many of which are novel and evolutionarily conserved, that can inform future studies.

## 4 METHODS

### 4.1 Cell culture

Yeast cells were grown in rich yeast extract peptone dextrose (YPD) media (1% (wt/vol) yeast extract, 2% (wt/vol) peptone, 2% (wt/vol) d‐glucose) or synthetic complete (SC) minimal media (0.675% (wt/vol) yeast nitrogen base without amino acids, 2% (wt/vol) d‐glucose, plus the appropriate amino acid or base dropouts for selection; (Formedium Norfolk, UK). Standard SC media contained 4 mg/L uracil and lower concentrations used, typically 0.1 and 0.05 mg/L for uracil stress conditions listed throughout. Cells were routinely cultured overnight to early/mid‐log phase (OD_600_ < 1.0) prior to experimental procedures. Yeast strains used in this study are listed in Table S7 and plasmids used are itemized in Table S8.

### 4.2 Yeast growth assays

For the primary screen, 10 μL culture of each mutant strain was grown in the well of a 96‐well plate containing 150 μL of YPD media with 250 μg/mL G418 overnight at 30°C in a humidified incubator. The bulk of YPD was then removed, followed by resuspension in water, and transfer of 10 μL to a fresh 96‐well plate containing 200 μL sterile water. Dilutions were then mixed with a 96‐pin replicator and pinned onto solid agar in 1536 format using a ROTOR‐HDA (Singer Instruments). Each mutant was pinned 16 times on solid media containing varying concentrations of uracil (4 or 0.1 or 0.05 mg/L) incubated at 30°C until sufficient growth was observed, followed by Phenobooth image capture (Singer Instruments) to record yeast growth. For follow‐up growth assays, the principle was the same, but cells were cultured in 5 mL serial dilutions to capture mid‐log phase cells, which were then harvested with equivalent volumes to other strains, including a wild‐type control, to be plated together. Six‐step serial dilutions (10‐fold) of each strain was generated in sterile water, followed by plating on SC plates containing of 4, 0.1 and 0.05 mg/L uracil. Plates were then incubated at 30°C for 3 days. Plates were imaged and the signal intensity of each spot was measured (ImageJ; NIH). Signal intensity of dilutions 3 to 5 were normalized to background and averaged and then values relative to WT were calculated.

### 4.3 Confocal microscopy

Yeast cells expressing GFP and/or mCherry tagged proteins were grown to mid‐log phase and then viewed in kill‐buffer (50 mM Tris pH 8.0, 10 mM NaN_3_, 10 mM NaF) or water (dH_2_O) at room temperature on Zeiss laser scanning confocal instruments (LSM710 or LSM880 equipped with Airyscan) with a Plan‐Apochromat 63×/1.4 Differential Interference Contrast (DIC) objective lens. Images were captured using Zen Black Imaging software and modified using Image J (NIH). Yeast vacuoles were labelled with 1.6 μM FM4‐64 (ThermoFisher) in YPD media, followed by 3× washes with SC media and further growth in SC media for 1‐hour prior to imaging. Where stated cells were fixed for imaging by spinning down mid‐log cultures and washing with 100 mM potassium phosphate buffer (pH 8) at 7000 rpm. Cells were resuspended and incubated for 10 min at room temperature in 4% paraformaldehyde (950 μL K‐Phos, 50 μL PFA) before spinning at 7000 rpm and resuspending in 1× PBS. Fixed cells were stained with Rhodamine‐phalloidin (Phalloidin‐594) and DAPI.

### 4.4 α‐factor‐induced arrest “halo” assay

Wild‐type and mutant Matα cells were grown to saturation overnight and then diluted back and grown for 4 to 6 hours in YPD media. Equivalent volumes of cells were harvested and spun down and brought up in 50 μL sterile water before spotting on low density lawns of MatA *bar1‐1* cells created from mid‐log phase cells on YPD plates. Plates were incubated at 30° overnight. Halos of growth inhibition for mutants relative to wild type were determined using ImageJ (NIH).

### 4.5 Gene ontology analyses

All GO enrichments were acquired using the GO Term Finder v0.86,^39^ using the default settings. The default background was used for all analyses except those for clusters derived from essential gene expression analysis, which instead used a background of essential genes listed in Table S4.

### 4.6 Hierarchical clustering and gene expression analyses

Microarray data documenting changes in gene expression in mutant strains lacking transcriptional regulators compared to wild type were assembled, representing 6123 genes. The data were read into R (v4.0.4; R Core Team, 2021) then processed using the dplyr v1.0.4^58^ and janitor v2.0.1^59^ packages to include only 28 strains lacking transcription factors identified in the uracil‐scavenging genetic screen. For downstream bioinformatic analyses, two gene reference subsets were generated: the first included all 147 verified candidates from the screen, and the second included 1183 genes denoted as essential for viability. For all analyses, hierarchical clustering was performed using the pheatmap package v1.0.12^60^ using complete linkage. Elbow and silhouette analyses were performed using the factoextra package v1.0.7^61^ to determine the optimal number of clusters to guide further GO enrichment analyses. The Pearson correlation matrix of the whole genome expression data was produced with base R and visualized with the pheatmap package.

### 4.7 String analysis

Interactome analysis of physical interactions was carried out using STRING software.^62^

### 4.8 Statistical analysis

Statistical significance for experimental conditions was calculated using a Student *t* test/Bonferroni‐Dunn method in GraphPad prism v8. Asterisks were used to denote significance on scatterplot histograms with *P* values documented in Tables S1 and S9.

## CONFLICT OF INTEREST

The authors declare no potential conflict of interest.

5

### PEER REVIEW

The peer review history for this article is available at https://publons.com/publon/10.1111/tra.12815.

## Supporting information


**Table S1** Uracil growth screen results and statistics

**Table S2** Orthologues of screen candidates and associated human diseases

**Table S3** GO enrichment annotations for biological processes

**Table S4** Genes essential for viability used in bioinformatics

**Table S5** Cluster analysis of essential genes regulated by screen TFs

**Table S6** Mating factor induced growth arrest screen results and statistics

**Table S7** Yeast strains used in this study

**Table S8** Plasmids used in this study

**Table S9** Statistical tests

**Appendix S1:** Supporting Information
