# Supplementary material for: Fur4‐mediated uracil‐scavenging to screen for surface protein regulators
Source: Traffic. 2021 Sep 23;22(11):397–408. doi: 10.1111/tra.12815 (PMC8650575; doi:10.1111/tra.12815)
Supplement: Supplementary file 10 — Appendix S1: Supporting Information [file TRA-22-397-s008.pdf]

SUPPLEMENTAL MATERIAL

Fur4 mediated uracil-scavenging to screen for surface protein trafficking regulators

SUPPLEMENTAL FIGURES AND LEGENDS

- **Figure S1:** Growth assay of known trafficking mutants.
- **Figure S2:** Methionine auxotroph trafficking mutants grow efficiently in low methionine media
- **Figure S3:** Interactome analysis of factors identified from screen
- **Figure S4:** Correlation matrix of responsive TF mutants of interest
- **Figure S5:** Expression of *YDR222W* in transcriptional mutants
- **Figure S6:** Fluorescently labelled surface cargoes
- **Figure S7:** Localisation effects of fluorescently tagging Tna1

SUPPLEMENTAL TABLES

- **Table S1:** Uracil growth screen results and statistics
- **Table S2:** Orthologues of screen candidates and associated human diseases
- **Table S3:** GO enrichment annotations for biological processes
- **Table S4:** Genes essential for viability used in bioinformatics
- **Table S5:** List of genes from clusters of downregulation
- **Table S6:** Mating factor induced growth arrest screen results and statistics
- **Table S7:** Yeast strains used in this study
- **Table S8:** Plasmids used in this study
- **Table S9:** Statistical significance tests

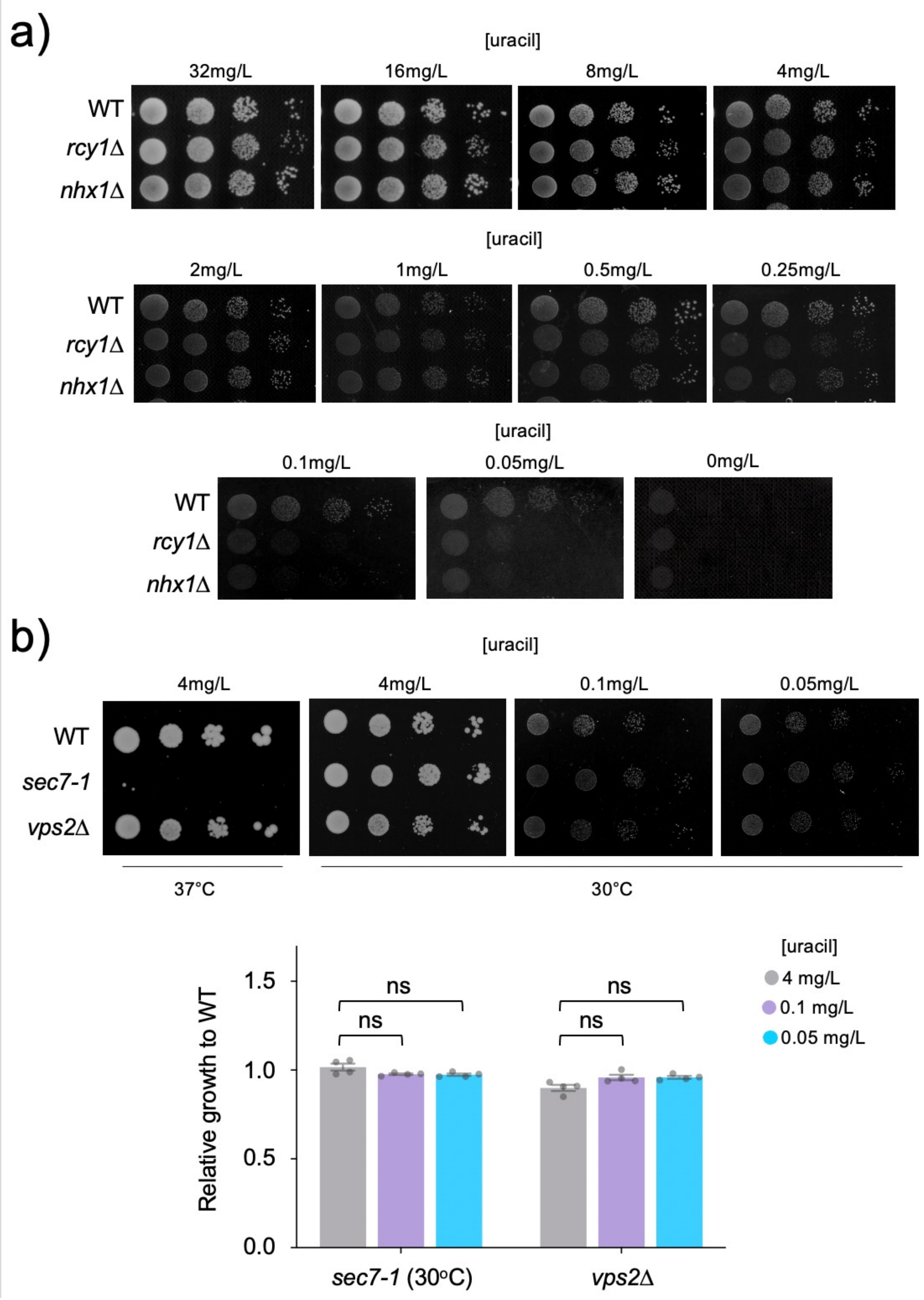

Figure S1: Growth assay of known trafficking mutants

a) Wild-type, *rcy1*Δ and *nhx1*Δ cells grown to mid-log phase were spotted in a 1 in 10 serial dilution onto plates of 32, 16, 8, 4, 2, 1, 0.5, 0.25, 0.1, 0.05 and 0 mg/L uracil and incubated at 30°C for 3 days.

b) Wild-type, *sec7-1* and *vps2*Δ cells were spotted onto plates of 4, 0.1 and 0.05 mg/L and incubated at 37°C or 30°C for 3 days. A students t-test (was carried out to determine significance).

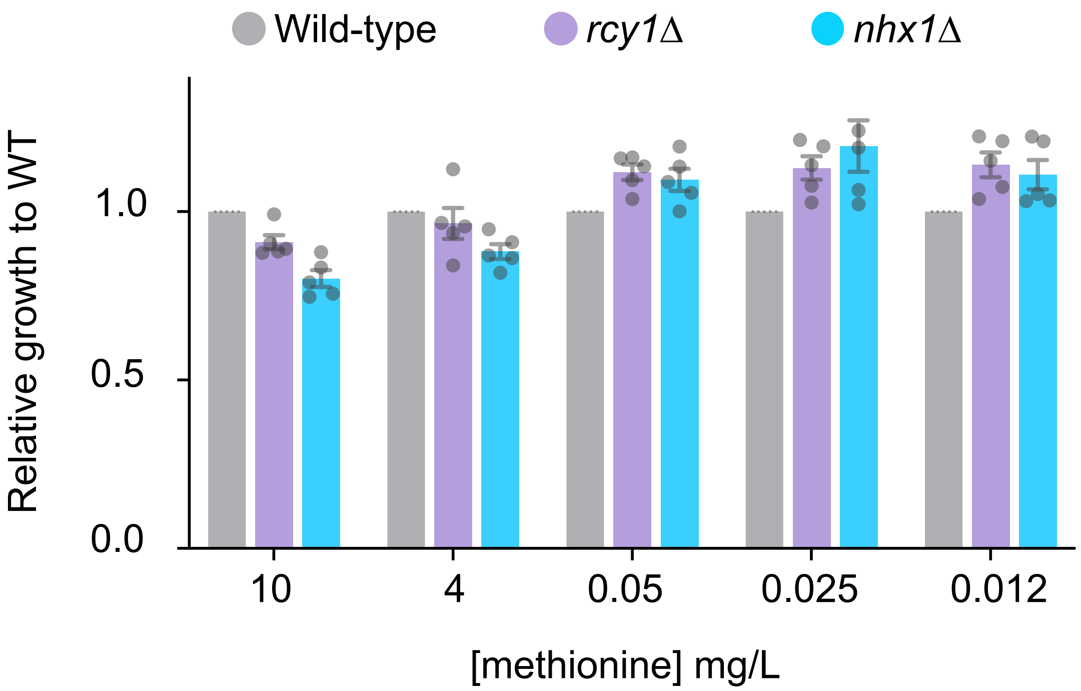

Figure S2: Methionine auxotroph trafficking mutants grow efficiently in low methionine media

Wild-type BY4741 Mat***A*** cells harbouring the *met15*Δ mutation that confers methionine auxotrophy, and two additional strains in this background additionally harbouring *rcy1*Δ (purple) or *nhx1*Δ (blue) mutations, were grown on SC media plates containing various indicated concentrations of methionine. There was no concentration of methionine that supported growth whilst also resulting in any significant defect in the strains with defective trafficking to the surface.

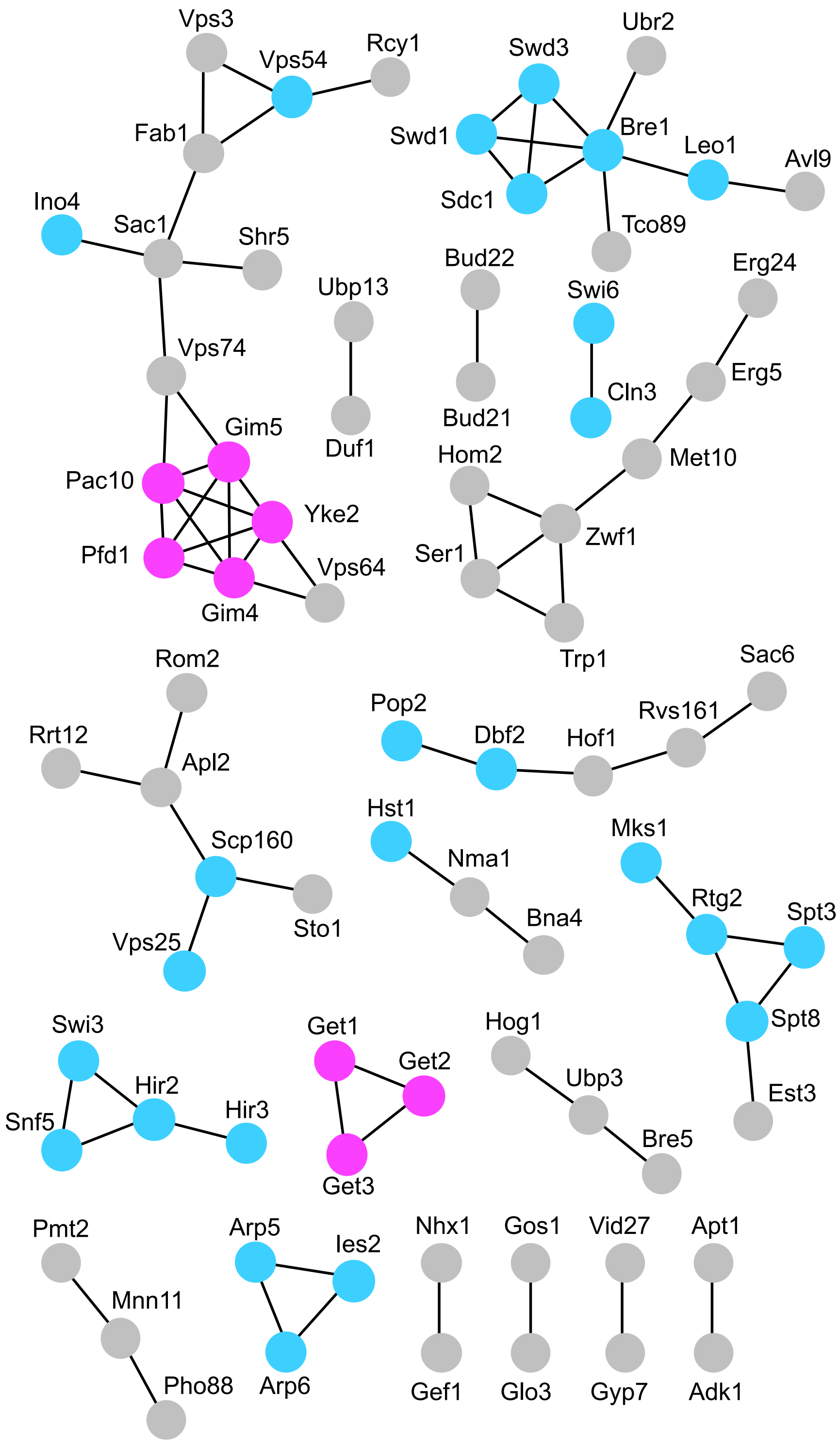

Figure S3: Interactome analysis

STRING pathway analysis for factors with known physical interactions identified in the low uracil-specific growth screen. Factors enriched for GO terms associated with cellular component (pink) and those that are included in bioinformatic assessment of gene expression upon their deletion (blue) are indicated. Orphan candidates with no interactions have been removed.

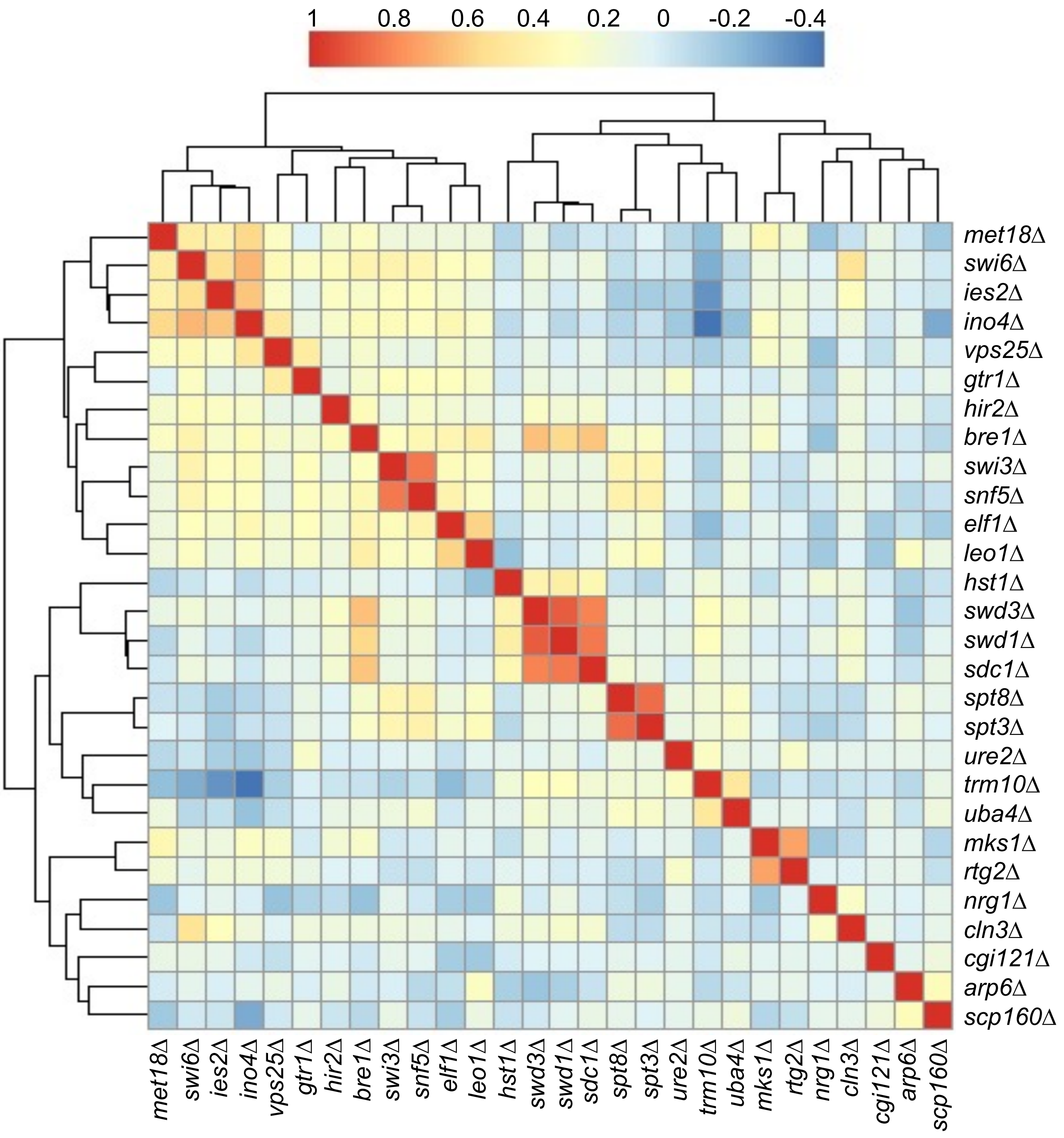

Figure S4: Correlation matrix of responsive TF mutants of interest

Correlation matrix of responsive mutants of interest, where 1 is perfect correlation as found from comparing identical mutants. Mutants of known interacting partners, for example Swd1, Swd3, Sdc1, exhibit high levels of correlation.

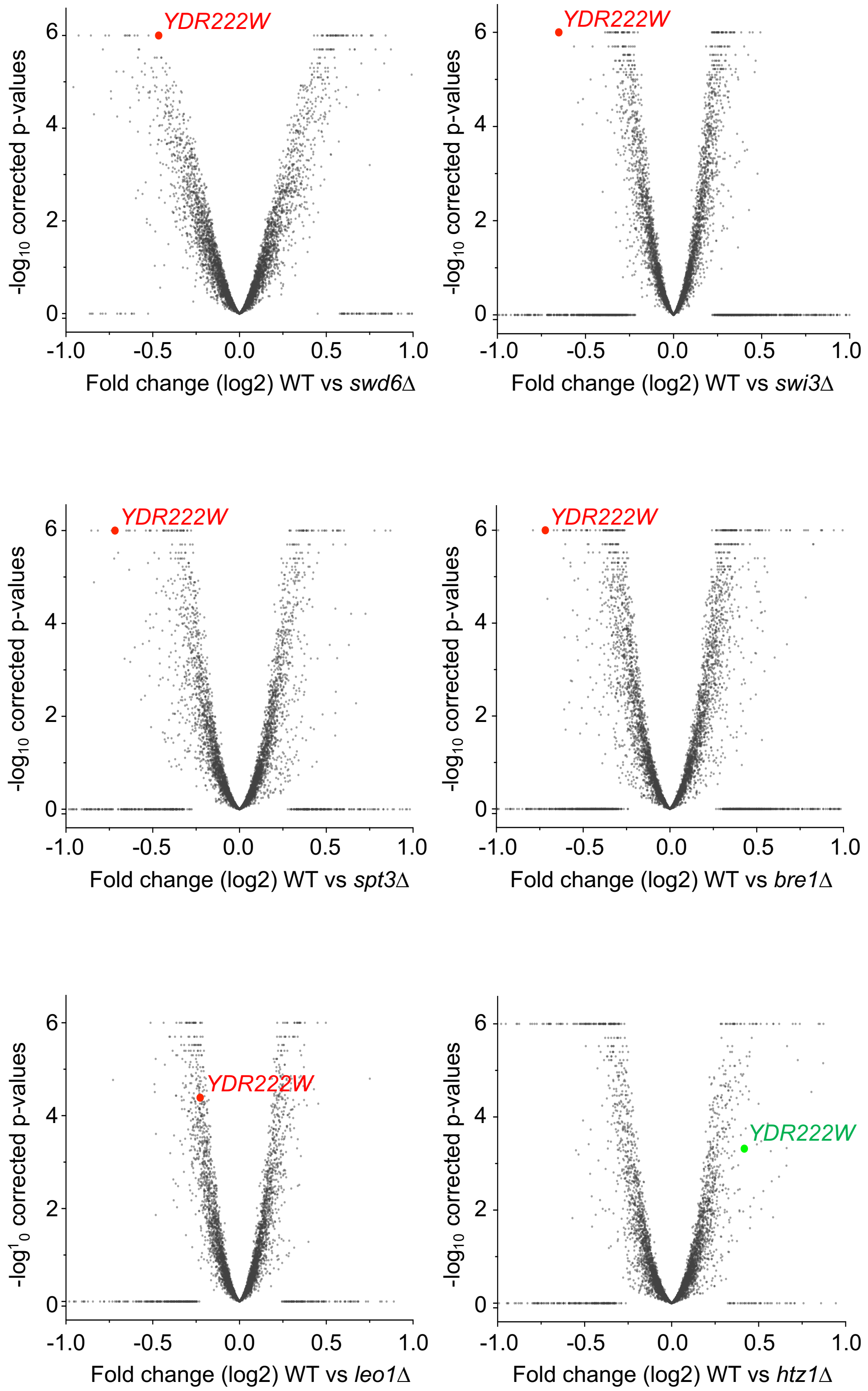

Figure S5: Expression of YDR222W in transcriptional mutants

Volcano plots were constructed for $\log_2$ fold changes and their corresponding p-values for genes in microarray analyses comparing wild-type cells to indicated mutants. Value for *YDR222W* expression is shown in each plot from mutants identified fro mthe screen (red) and an independent analysis of *htz1Δ* mutants that were not identified as Fur4-related from the screen (green).

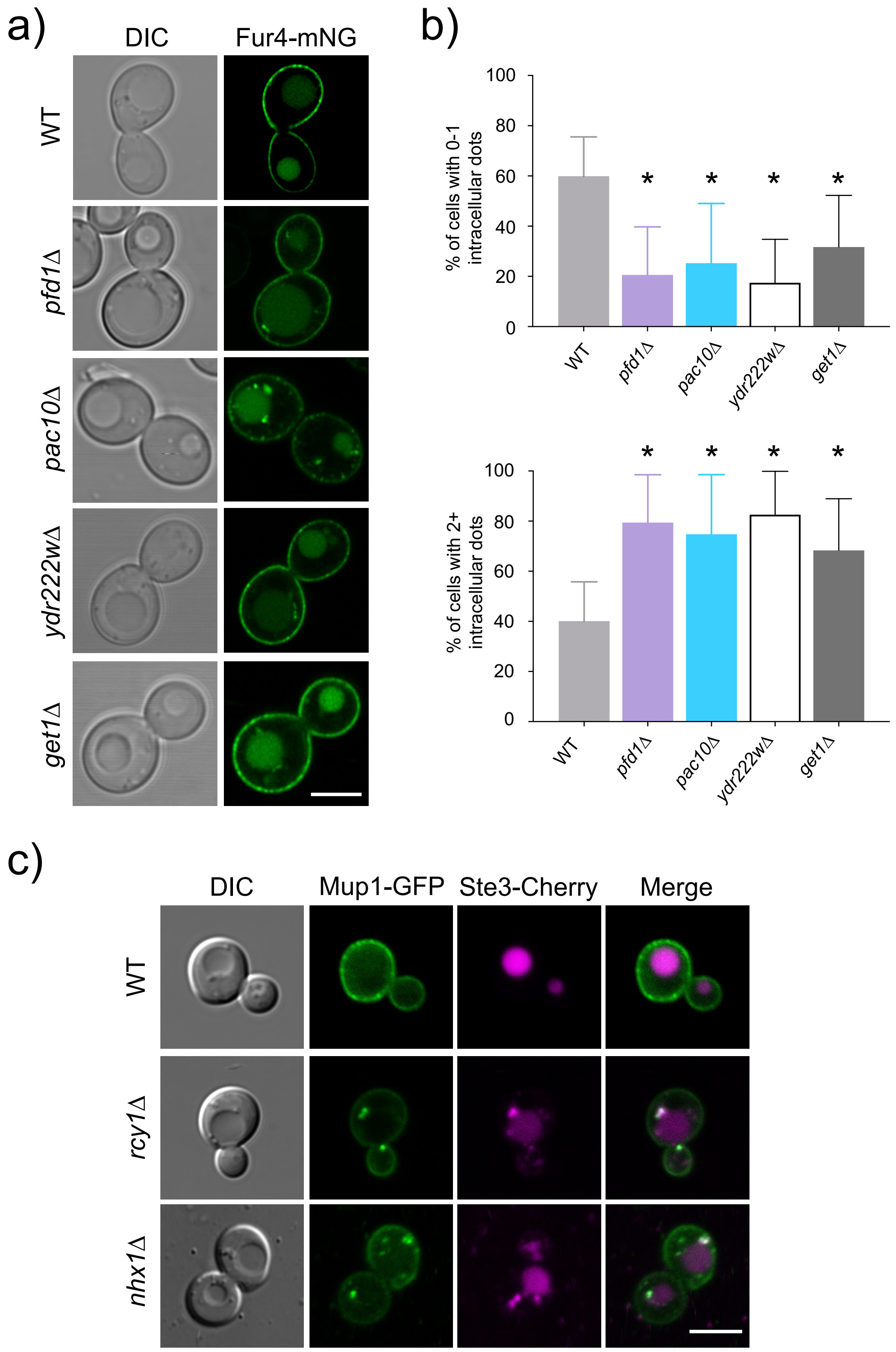

Figure S6: Fluorescently labelled surface cargoes

a) Fur4-mNeonGreen localisation in indicated strains.
b) Percentage of cells with either 0-1 (upper) or 2+ (lower) intracellular puncta were quantified (n = >30).
c) Indicated cells were co-expressing Mup1-GFP and Ste3-mCherry under control of endogenous promoters were grown to mid-log phase and imaged by confocal microscopy.

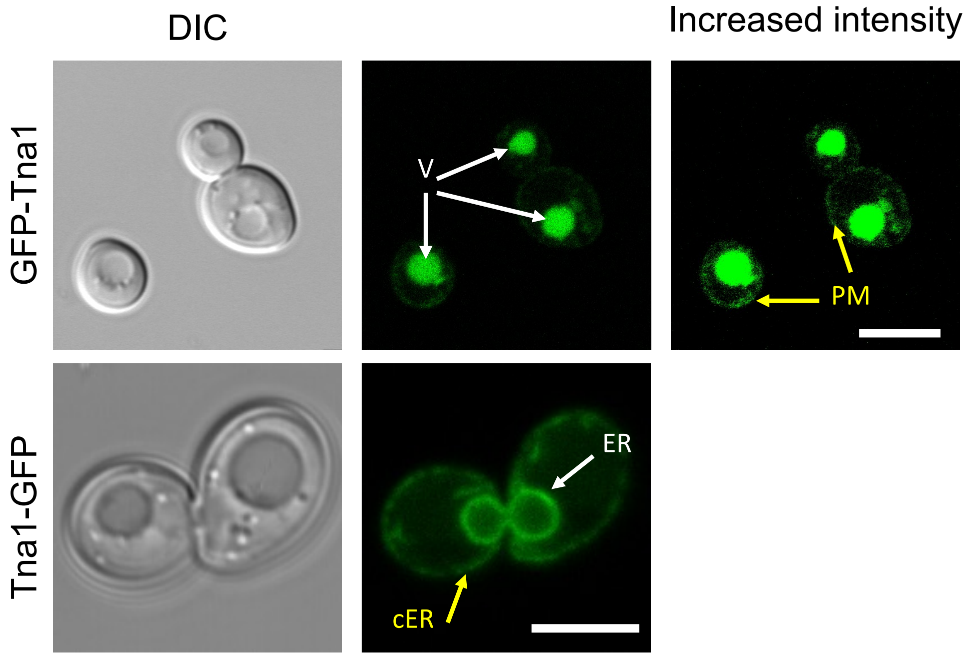

Figure S7: Localisation effects of fluorescently tagging Tna1

Wild-type cells expressing either N-terminally (GFP-Tna1) or C-terminally (Tna1-GFP) GFP tagged Tna1 were imaged at log phase. PM = Plasma Membrane, cER = cortical Endoplasmic Reticulum and ER = Endoplasmic Reticulum. Scale bar = 5μm.

SUPPLEMENTAL REFERENCES

1. Brachmann, C. B. *et al. Designer deletion strains derived from Saccharomyces cerevisiae S288C: A useful set of strains and plasmids for PCR-mediated gene disruption and other applications*. *Yeast* **14**, (1998).
2. Breslow, D. K. *et al.* A comprehensive strategy enabling high-resolution functional analysis of the yeast genome. *Nat. Methods* **5**, 711–718 (2008).
3. Ciejek, E. & Thorner, J. Recovery of S. cerevisiae a cells from G1 arrest by α factor pheromone requires endopeptidase action. *Cell* **18**, 623–635 (1979).
4. Laidlaw, K. M. E. *et al.* A glucose-starvation response governs endocytic trafficking and eisosomal retention of surface cargoes in budding yeast. *J. Cell Sci.* **134**, jcs.257733 (2021).
5. Winzeler, E. A. *et al.* Functional characterization of the S. cerevisiae genome by gene deletion and parallel analysis. *Science (80-.).* **285**, 901–906 (1999).
6. MacDonald, C. *et al.* A Family of Tetraspans Organizes Cargo for Sorting into Multivesicular Bodies. *Dev. Cell* **33**, 328–342 (2015).
7. Giaever, G. *et al.* Functional profiling of the Saccharomyces cerevisiae genome. *Nature* **418**, 387–391 (2002).
8. Dowell, R. D. *et al.* Genotype to phenotype: A Complex problem. *Science* **328**, 469 (2010).
9. Rabitsch, K. P. *et al.* A screen for genes required for meiosis and spore formation based on whole-genome expression. *Curr. Biol.* **11**, 1001–1009 (2001).
10. Kastenmayer, J. P. *et al.* Functional genomics of genes with small open reading frames (sORFs) in S. cerevisiae. *Genome Res.* **16**, 365–373 (2006).
11. Bloom-Ackermann, Z. *et al.* A Comprehensive tRNA Deletion Library Unravels the Genetic Architecture of the tRNA Pool. *PLoS Genet.* **10**, (2014).
